# Carbon Dioxide Enrichment Partially Alleviates the Impact of Drought Stress on Cotton Growth and Yield

**DOI:** 10.3390/plants15142189

**Published:** 2026-07-17

**Authors:** Naflath Thenveettil, Manoj Kumar Reddy Allam, Krishna N. Reddy, Michael Cox, Wei Gao, Kambham Raja Reddy

**Affiliations:** 1 Department of Plant and Soil Sciences, Mississippi State University, 117 Dorman Hall, P.O. Box 9555, Mississippi State, MS 39762, USA; ma2129@msstate.edu (M.K.R.A.); mcox@pss.msstate.edu (M.C.); 2 USDA-ARS Crop Production Systems Research Unit, 141 Experiment Station Road, P.O. Box 350, Stoneville, MS 38776, USA; krishna.reddy@usda.gov; 3 USDA UVB Monitoring and Research Program, Department of Ecosystem Science and Sustainability, Colorado State University, Fort Collins, CO 80523, USA; wei.gao@colostate.edu

**Keywords:** boll production, boll maturation period, yield, fiber quality, physiology

## Abstract

This study investigated the effects of drought during reproductive and boll development stages under elevated CO_2_ conditions. A pot experiment was conducted in the Soil–Plant–Atmospheric-Research (SPAR) facility using the upland cotton cultivar DP 1646 B2XF grown under control (well-watered; 0.12 m^3^ H_2_O m^−3^ soil) and drought (0.09 m^3^ H_2_O m^−3^ soil) conditions at ambient (425 ppm; aCO_2_) and enriched (725 ppm; eCO_2_) CO_2_ concentrations. Under drought stress, photosynthesis and stomatal conductance decreased by 35% and 63%, respectively, under aCO_2_, whereas reductions were less pronounced under eCO_2_ (20% and 36%). Under drought and aCO_2_, intrinsic and instantaneous water-use efficiency increased by 74% and 45%, respectively, compared to control. Biomass partitioning shifted under drought, with increased allocation to shoots (55%) and roots (9%) and reduced allocation to reproductive organs (36%), compared to control conditions. Flowers produced under drought had 21% fewer ovules under both CO_2_ environments, while pollen production remained unaffected. Seed cotton and lint weights were reduced by 19% and 15% under drought, respectively. However, plants grown under eCO_2_ attained seed cotton and lint weights that were 20% higher than those grown under aCO_2_, under both control and drought conditions. Drought significantly affected fiber quality, increasing micronaire by 26% and reducing fiber length by 4%, regardless of CO_2_ level. eCO_2_-driven alleviation primarily acts through carbon assimilation and yield compensation, with limited capacity to regulate developmental programming and quality formation.

## 1. Introduction

Drought is a major abiotic stress that greatly limits plant growth, development, and productivity, posing a serious challenge for global agriculture [1,2]. It is defined as a prolonged deficiency in precipitation, typically lasting a season or more, resulting in a water shortage [3,4]. Water is a crucial component of fresh biomass, making up about 80–95% of plant biomass, and is vital for various aspects of plant growth and metabolism. Over the past decades, droughts have become more frequent and severe in the US, leading to significant economic impacts [5]. Recent drought assessments indicate that most cropland is affected, resulting in severe economic losses and detrimental effects on ecosystems [6,7]. Cotton (*Gossypium hirsutum* L.), a globally important fiber crop, is particularly vulnerable to episodic and prolonged water deficits because reproductive development and fiber formation are highly sensitive to plant water status [8,9,10]. In major cotton-producing regions of the United States, recurrent drought and heat stress have contributed to yield instability, reduced harvested area, and substantial economic losses [6,7]. Drought impacts crop yields and harvested areas, causing reductions of 9–10% under moderate conditions and up to 85% in severe cases [11,12].

At the plant level, drought reduces leaf water potential and stomatal conductance, thereby limiting CO_2_ uptake for photosynthesis [2,12,13,14]. Drought stress particularly affects photosystem II due to its high sensitivity to water deficits and its instability during electron transport [15]. Under drought conditions, there is often an increase in the activities of antioxidant enzymes, such as superoxide dismutase and catalase, as well as an accumulation of osmolytes such as sugars and proline [16,17]. Additionally, drought stress disrupts nutrient uptake and transport, alters hormonal balance, particularly through abscisic acid-mediated stomatal closure, and can increase oxidative stress [18,19,20]. These physiological disruptions result in stunted growth, reduced leaf area, and delayed development, particularly during critical growth stages such as flowering and boll formation [21]. Water shortages at this stage can lead to decreased flower production, increased flower/boll abortion, and fewer bolls setting, all of which significantly reduce overall yield [22]. Additionally, drought stress during boll development can exacerbate yield losses by producing smaller bolls and fewer seeds [12,23,24]. It can also negatively impact economically important fiber quality traits, such as fiber length, strength, and uniformity [8,10].

Rising atmospheric CO_2_ concentration is expected to alter crop responses to drought. Elevated CO_2_ (eCO_2_) commonly enhances photosynthesis and can improve intrinsic water-use efficiency through partial stomatal closure, which may lessen some drought-related constraints on plant carbon balance and productivity [25,26,27]. In cotton, several studies have examined the effects of drought and eCO_2_ on growth and physiology, including canopy photosynthesis and water-use efficiency, as well as interactions with additional environmental factors, such as warming [28,29]. These studies have substantially advanced the understanding of physiological responses, but they also highlight that eCO_2_-mediated mitigation is context-dependent and may not translate uniformly into reproductive outcomes and crop quality.

A key unresolved question is how physiological responses to drought and eCO_2_ relate to reproductive dynamics (boll production and retention), final yield components, and fiber quality traits, particularly when temperature is held constant, allowing CO_2_ × water effects to be evaluated without confounding thermal stress. This distinction is important because partial recovery of photosynthesis or water-use efficiency under eCO_2_ does not necessarily imply proportional recovery of boll retention, lint yield, or fiber quality.

Therefore, the objective of this study was to quantify the combined effects of drought and eCO_2_ on cotton across multiple response scales at constant temperature. Specifically, we aimed to: (i) determine how gas exchange and water-use efficiency respond to drought and eCO_2_, (ii) evaluate effects on boll production and retention dynamics, and (iii) assess consequences for seed and lint yield and fiber quality. By linking leaf-level physiology with reproductive performance and market-relevant fiber traits, this study provides an integrated assessment of cotton response to drought under eCO_2_.

## 2. Results

### 2.1. Drought and eCO_2_ Interaction on Gas Exchange and Water Potential

**Figure 1:**
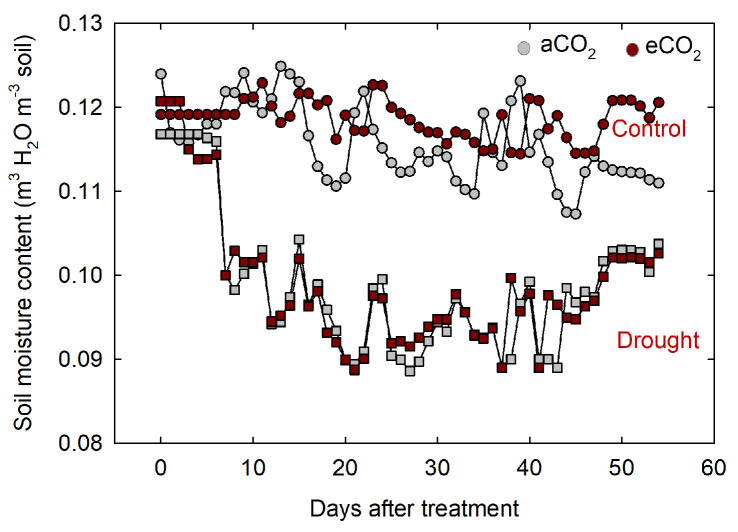
Temporal volumetric soil moisture content under control and drought conditions at ambient (425 ppm) and enriched (725 ppm) CO_2_ levels.

**Figure 2:**
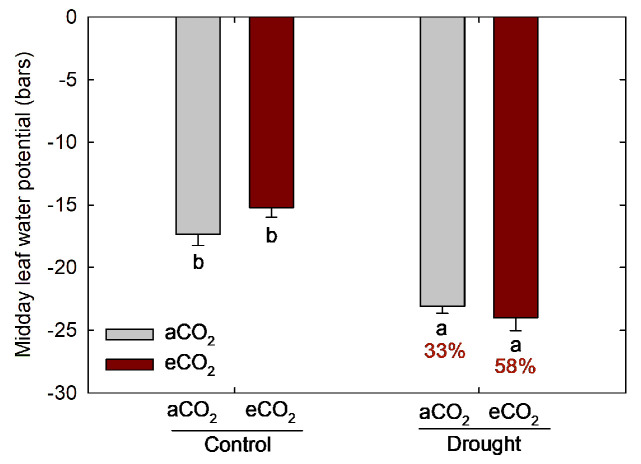
Midday leaf water potential (bars) of cotton under control and drought conditions at ambient (aCO_2_; 425 ppm) and enriched (eCO_2_; 725 ppm) CO_2_ levels. The bars represent the mean ± SE from three replicates. The letter above the bar indicates significance at *p* < 0.05. Treatments with the same letters are not significantly different. The percentage values above the bars are the percent differences between control and drought conditions under aCO_2_ and eCO_2_.

The average soil moisture content under the control and drought treatments was 0.12 and 0.09 m^3^ m^−3^, respectively, indicating a statistically significant difference at *p* < 0.05 (Figure 1). Lower soil moisture content in the drought treatment limited leaf water content, resulting in 33% and 58% decreases (high negative values) in midday leaf water potential under aCO_2_ and eCO_2_, respectively, compared to the control at the respective CO_2_ levels (Figure 2).

**Figure 3:**
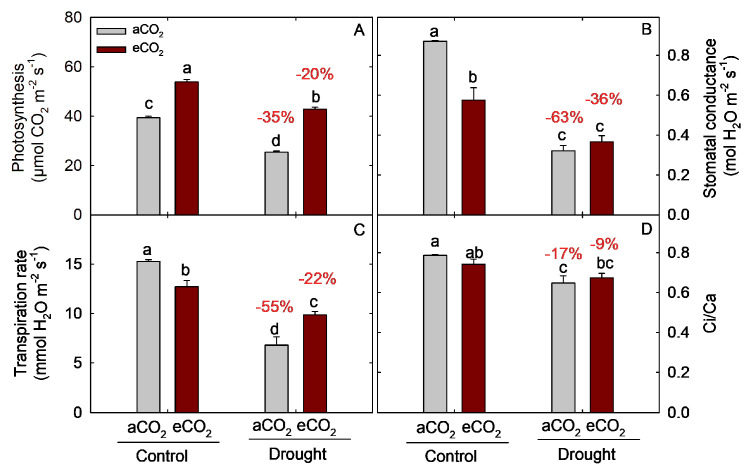
Gas exchange parameters ((**A**): Photosynthesis; (**B**): Stomatal conductance; (**C**): Transpiration; (**D**): Ci/Ca) of cotton under control and drought conditions at ambient (aCO_2_; 425 ppm) and enriched (eCO_2_; 725 ppm) CO_2_ levels. The bars represent the mean ± SE from three replicates. The letter above the bar indicates significance at *p* < 0.05. Treatments with the same letters are not significantly different. The percentage values above the bars are the percent differences between control and drought conditions under aCO_2_ and eCO_2_.

Cotton exhibited significant physiological responses to drought and eCO_2_ treatments during the reproductive stage. All the gas exchange measurements exhibited statistically significant changes under drought and eCO_2_ conditions. However, the Ci/Ca ratio did not change with eCO_2_ (Table 1). The interaction between drought treatment and eCO_2_ resulted in significant changes in gsw and transpiration rate (*p* < 0.01–0.001). The plants subjected to drought conditions showed a lower photosynthetic rate under both aCO_2_ (35%) and eCO_2_ (20%) conditions compared to the control (Figure 3A). However, eCO_2_ enhanced the photosynthetic rate by 37% and 68% under control and drought conditions, respectively, compared to aCO_2_. Reduced water availability under drought conditions decreased GSW by 63% and 36% under aCO_2_ and eCO_2_ conditions, respectively, compared to the control (Figure 3B). It is essential to note that the gsw decreased by 33% when grown under control + eCO_2_ conditions compared to control + aCO_2_. At the same time, it did not undergo significant changes under drought and eCO_2_ conditions. The reduction culminated in a decline in transpiration (Figure 3C). Under aCO_2_ and eCO_2_ conditions, transpiration was reduced by 55% and 22%, respectively, compared to the control at similar CO_2_ levels.

**Figure 4:**
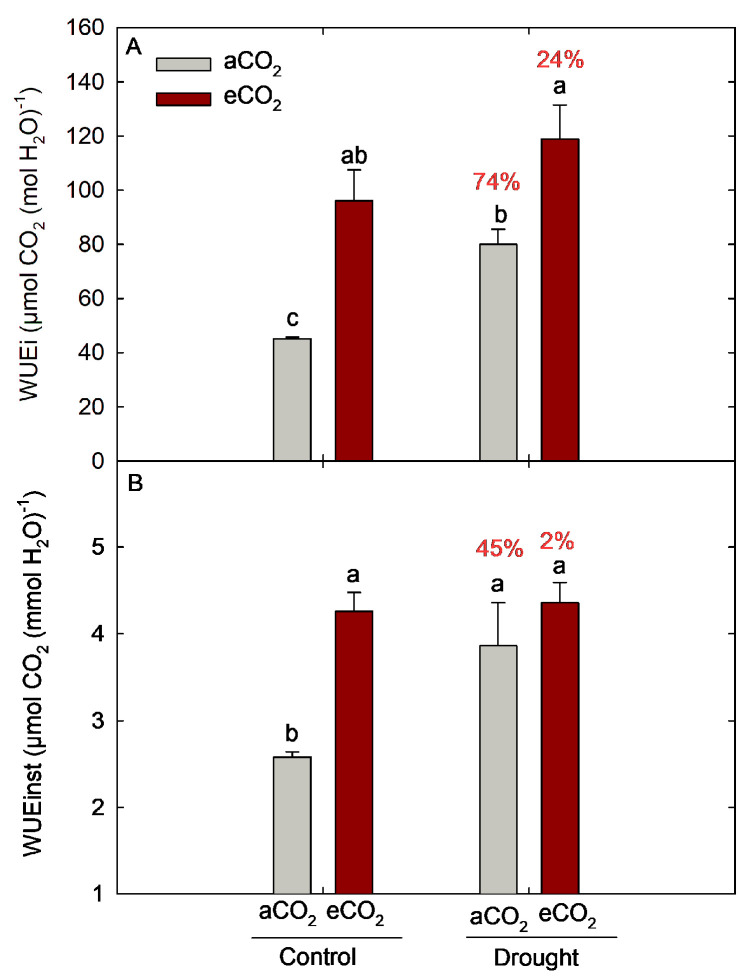
Water-use efficiency ((**A**): WUEi (Intrinsic water-use efficiency); (**B**): WUEinst (Instantaneous water-use efficiency)) of cotton under control and drought conditions at ambient (aCO_2_; 425 ppm) and enriched (eCO_2_; 725 ppm) CO_2_ levels. The bars are the mean ± SE of four replications. The letter above the bar indicates significance at *p* < 0.05. Treatments with the same letters are not significantly different. The percentage values above the bars are the percent differences between control and drought conditions under aCO_2_ and eCO_2_.

The WUEi and WUEinst of plants enhanced under aCO_2_ + drought conditions by 74% and 45%, respectively, compared to aCO_2_ + control (Figure 4A,B). Conversely, under eCO_2_, both water-use efficiency indices showed no significant difference between the control and drought treatments. On the other hand, plants exposed to eCO_2_ under control conditions showed increases of 107% and 48% in WUEi and WUEinst, respectively. Under drought stress, only WUE_i_ showed a significant eCO_2_-induced enhancement, increasing by 64%.

### 2.2. Drought Retarded Growth and Biomass in Cotton

**Figure 5:**
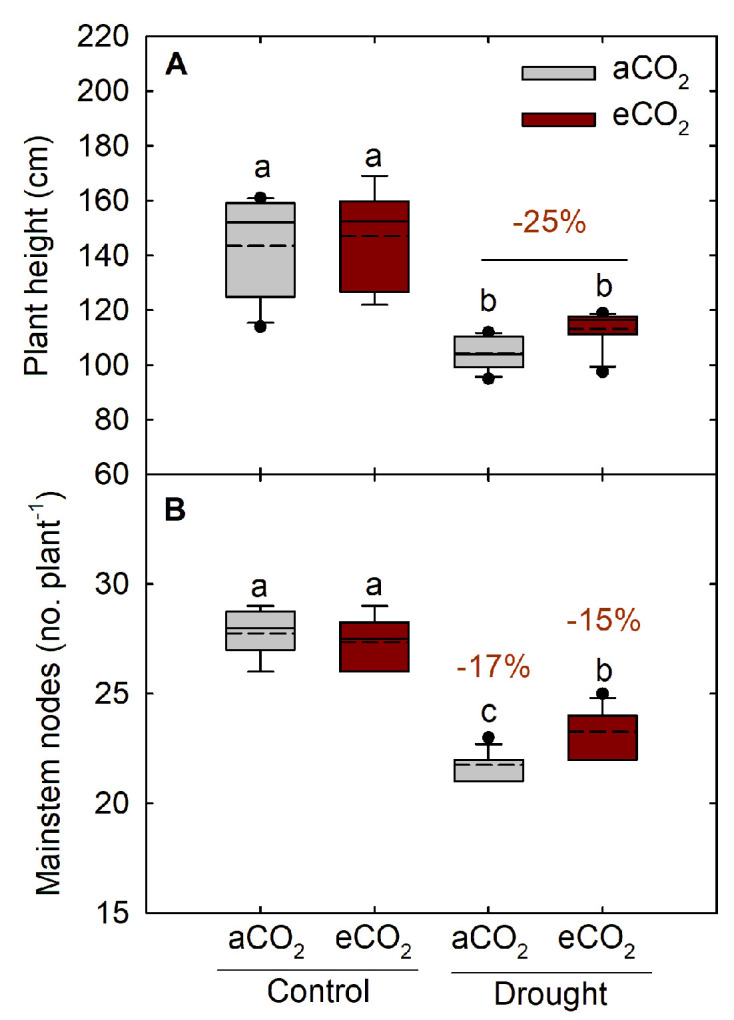
(**A**) Plant height and (**B**) mainstem node number of cotton under control and drought conditions at ambient (aCO_2_; 425 ppm) and enriched (eCO_2_; 725 ppm) CO_2_ levels. The boxes show the mean ± SE from 12 replications. The letter above the box indicates significance at *p* < 0.05. Treatments with the same letters are not significantly different. The percentage values above the bars are the percent differences between control and drought conditions under aCO_2_ and eCO_2_.

The drought treatment significantly influenced all the plant growth and biomass parameters (*p* < 0.01–0.001) (Table 1). eCO_2_ did not significantly influence growth and biomass, whereas the CO_2_–drought interaction was observed in the number of mainstem nodes. The height of the plants exposed to drought conditions under both aCO_2_ and eCO_2_ during the flowering and boll development stage declined by 25% (Figure 5A and Figure S1). Likewise, fewer mainstem nodes were recorded under drought + aCO_2_ conditions (22), followed by drought + eCO_2_ (23), than under control conditions (27; Figure 5B).

**Figure 6:**
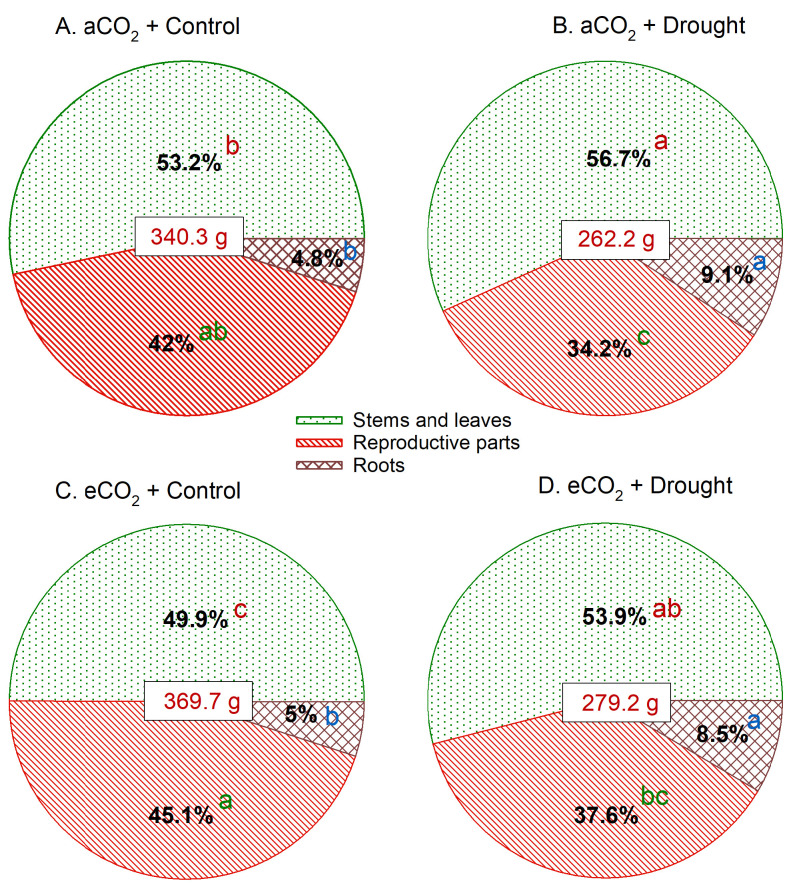
Total biomass and biomass partitioning (percentage of each organ out of total biomass) of cotton under (**A**,**C**) control and (**B**,**D**) drought conditions at ambient (aCO_2_; 425 ppm) and enriched (eCO_2_; 725 ppm) CO_2_ levels. The value in the white box is the total weight in grams. The red, green, and blue letters beside the percentage values indicate the statistical significance of the treatments for stem and leaves, reproductive part, and root biomass proportions, respectively.

The reductions in plant height and number of nodes under drought conditions significantly contributed to the overall decline in biomass accumulation (Figure 6). Total biomass of plants grown under aCO_2_ + drought conditions decreased by 23% compared to those under aCO_2_ + control (Figure 6A,B). Similarly, plants subjected to eCO_2_ + drought exhibited a 24% reduction in total biomass relative to the eCO_2_ + control (Figure 6C,D). Drought stress also altered biomass allocation patterns, notably increasing the proportion of root biomass. Under drought conditions, root biomass accounted for 9.1% (aCO_2_) and 8.5% (eCO_2_) of the total biomass, compared to 4.8% and 4.97% in the respective control treatments. Reproductive biomass (including squares and bolls) constituted only 34.2% (aCO_2_) and 37.5% (eCO_2_) of the total biomass under drought, markedly lower than the 42% and 45% recorded under control conditions at aCO_2_ and eCO_2_, respectively. Conversely, the proportion of shoot biomass (stems and leaves) increased under drought stress by approximately 6% (aCO_2_) and 8% (eCO_2_) compared to control plants.

### 2.3. Reproductive Performance of Plants Under eCO_2_ and Drought

**Figure 7:**
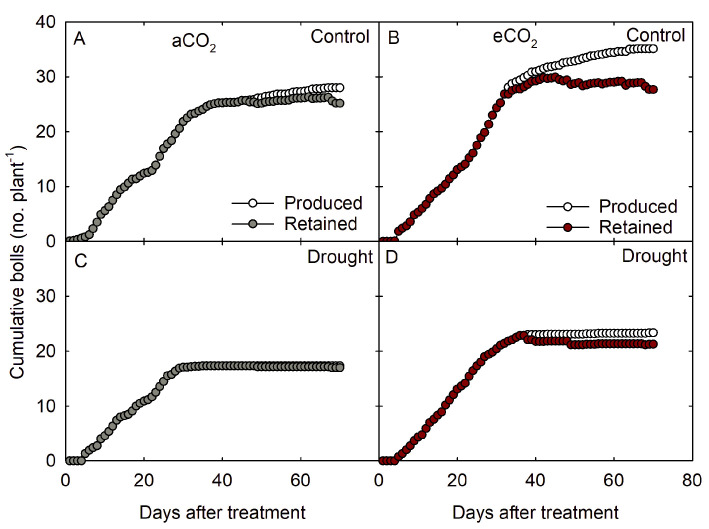
Cumulative number of bolls produced and retained at aCO_2_ (425 ppm) and eCO_2_ (725 ppm) concentrations under (**A**,**B**) control and (**C**,**D**) drought conditions.

Drought and enriched CO_2_ conditions modified boll production and retention in cotton. The per-plant cumulative boll production and retention under control and drought conditions at aCO_2_ and eCO_2_ are presented in Figure 7. The plants grown under aCO_2_ + control produced bolls continuously at a constant rate from 5 days after treatment (DAT) to 32 DAT, with an addition rate of 0.8 boll per day (Figure 7A). Beyond 32 DAT, boll production ceased, with only 5 more bolls by the end of the experiment. Under these conditions, the plants retained nearly all the bolls, dropping only 3 bolls by the end of the experiment. However, under eCO_2_ + control conditions, the boll production rate was around 0.9 bolls per day from 5 DAT up to 33 DAT (Figure 7B). While it reduced to 0.2 bolls per day from 34 DAT, producing only 7 more bolls by the end of the experiment. Under these conditions, boll drop was observed starting at 33 DAT, with 7 bolls lost by 70 DAT. Plants exposed to drought conditions produced fewer bolls than the control (Figure 7C,D). Under aCO_2_ + drought conditions, the plants produced 17 bolls by 30 DAT, with an additional rate of 0.6 bolls per day (Figure 7C). Beyond 30 DAT, the plants did not produce any additional bolls. Interestingly, all the bolls were retained throughout the experimental period. The eCO_2_ conditions under drought positively influenced boll production by increasing the boll production rate to 0.75 bolls per day from 5 DAT to 31 DAT (Figure 7D). The rate of boll production beyond 32 DAT diminished, incorporating only 3 more bolls by the end of the experiment. Of the 23 bolls produced under eCO_2_ + drought, the plants retained 21 by 70 DAT. As boll tagging was discontinued after 70 DAT, boll production and retention beyond this period were not monitored.

The number of open bolls and boll retention was recorded at the time of harvest. The number of open bolls was not influenced by eCO_2_ in either control or drought conditions, but it declined under drought (Table 2). The average number of open bolls was 25 under control conditions, and this decreased to 17.2 under drought stress. It is interesting to note that boll retention under aCO_2_ + drought conditions (97.9%) was higher than under aCO_2_ + control conditions (92.9%), despite the adverse effects on overall boll production. A reduction in BMP was recorded under drought stress at both CO_2_ conditions. Under aCO_2_ + drought, the bolls matured in 45.9 days compared to 47.3 days under control. Under eCO_2_ + drought, bolls matured in 44.7 days, compared to 47.1 days under control. It is worth noting that boll maturation further accelerated under eCO_2_ + drought stress conditions, but eCO_2_ alone did not alter maturation timing.

**Figure 8:**
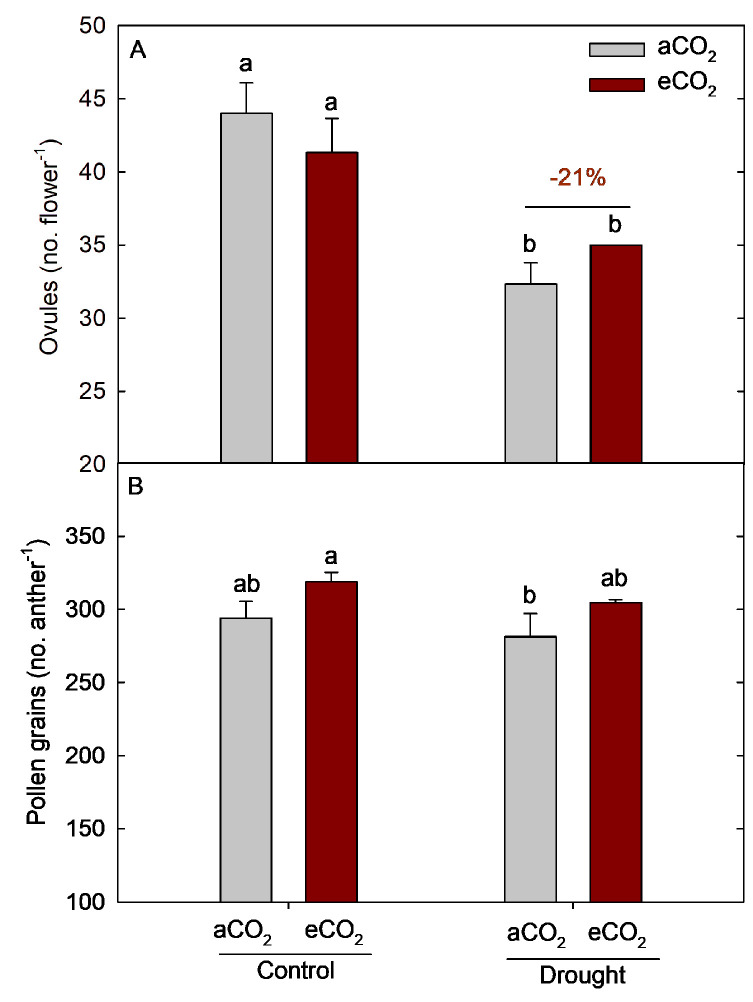
Number of (**A**) ovules and (**B**) pollen grains of cotton under control and drought conditions at ambient (aCO_2_; 425 ppm) and enriched (eCO_2_; 725 ppm) CO_2_ levels. The bars represent the mean ± SE from three replicates. The letter above the bar indicates significance at *p* < 0.05. Treatments with the same letters are not significantly different. The percentage values above the bars are the percent differences between control and drought conditions under aCO_2_ and eCO_2_.

**Figure 9:**
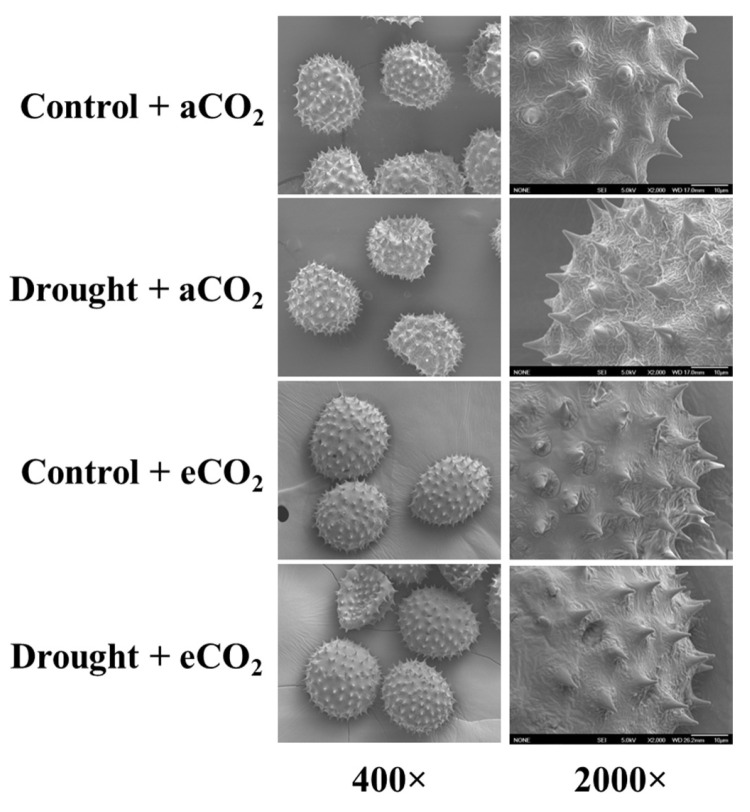
Pollen grain morphology of cotton under control and drought conditions at ambient (aCO_2_; 425 ppm) and enriched (eCO_2_; 725 ppm) CO_2_ levels. The figures are magnified at 400× and 2000×.

The number of ovules (no. flower^−1^) and pollen grains (no. anther^−1^) were recorded under drought and CO_2_ conditions. The drought conditions significantly influenced the number of ovules (*p* < 0.001), whereas eCO_2_ did not affect either ovule or pollen grain production (Table 1; Figure 8A,B). Ovule production was significantly suppressed by drought stress, resulting in an average 21% reduction under both CO_2_ conditions (Figure 8A). Pollen grains produced under drought conditions appeared largely intact and comparable to those from control conditions, with only minor shape abnormalities observed in a few grains (Figure 9).

### 2.4. Seed Yield and Fiber Quality Under Drought and CO_2_ Conditions

**Figure 10:**
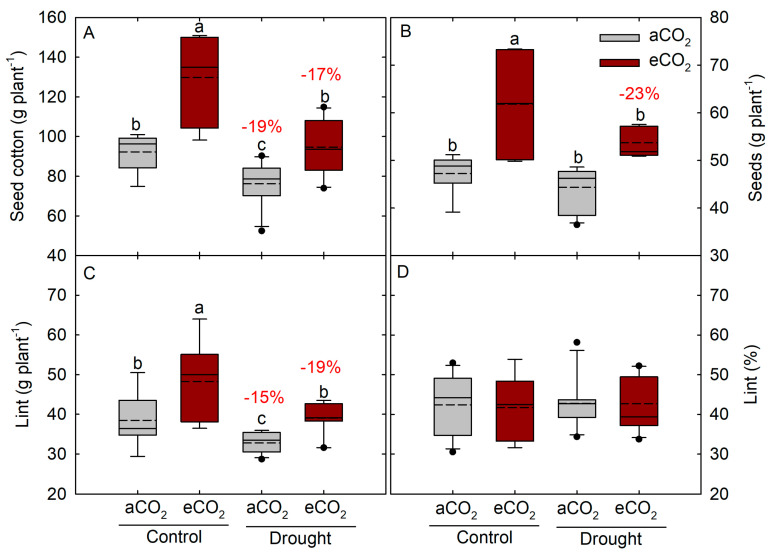
(**A**) Seed cotton, (**B**) seed, (**C**) lint weights, and (**D**) lint (%) of cotton under control and drought conditions at ambient (aCO_2_; 425 ppm) and enriched (eCO_2_; 725 ppm) CO_2_ levels. The bars are the mean ± SE of six replications. The letter above the bar indicates significance at *p* < 0.05. Treatments with the same letters are not significantly different. The percentage values above the bars are the percent differences between control and drought conditions under aCO_2_ and eCO_2_.

The seed and lint yields of the plants were significantly affected by eCO_2_ (*p* < 0.01–0.001) and drought (*p* < 0.01–0.001) conditions (Table 1). The interaction between CO_2_ and drought was non-significant. Seed cotton and lint weight declined due to drought stress under both CO_2_ conditions. Seed cotton yield decreased by 19% under aCO_2_ and by 17% under eCO_2_, while lint yield decreased by 15% and 19%, respectively, compared to the control (Figure 10A,C). On the other hand, the eCO_2_ conditions enhanced seed cotton yield by 21% under control conditions and by 24% under drought, while lint yield increased by 25% and 19%, respectively. Conversely, seed yield remained unaffected by drought stress under aCO_2_ conditions, whereas under eCO_2_ it showed a 23% reduction (Figure 10B). Lint (%) was unaffected by either drought or CO_2_ conditions.

**Figure 11:**
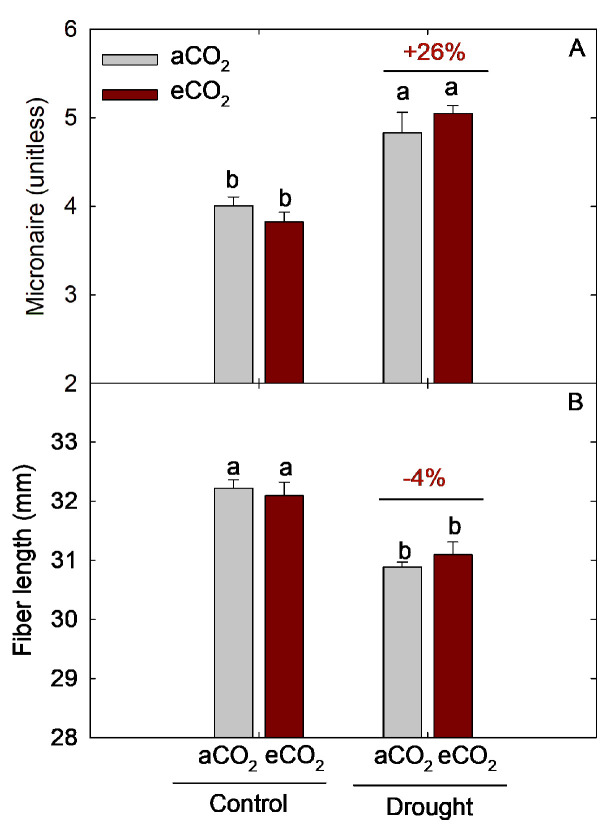
(**A**) Micronaire and (**B**) fiber length of cotton under control and drought conditions at ambient (aCO_2_; 425 ppm) and enriched (eCO_2_; 725 ppm) CO_2_ levels. The bars are the mean ± SE of four replications. The letter above the bar indicates significance at *p* < 0.05. Treatments with the same letters are not significantly different. The percentages above the bars are the average percent differences between the control and drought conditions.

The fiber quality parameters, such as micronaire, fiber length, uniformity, and strength, did not change due to eCO_2_, whereas drought significantly affected micronaire and fiber length (*p* < 0.001; Table 1). The interaction between drought and CO_2_ was not significant for any of the recorded fiber quality parameters. Fibers produced under drought conditions increased micronaire by 26% while shortening length by 4% (Figure 11A,B).

## 3. Discussion

Water is the most important factor affecting plant growth and development. Sub-optimal water levels limit plants’ physiological and biochemical processes, affecting the overall plant growth and homeostasis [30]. The study observed alterations in the plant’s reproductive performance, biomass accumulation, yield, and cotton fiber quality. The non-significant interaction between drought and CO_2_ could be due to the smaller sample size. The drought stress-induced reduction (negative) in leaf water potential afflicts the plant hydraulic transport system, as xylem embolism and associated processes in xylem tend to increase the hydraulic resistance along the soil–plant–atmosphere continuum [31]. This leads to a cessation of water transport in the plant system, resulting in desiccation of tissues and organs during extreme events [32]. However, as an adaptive mechanism to drought stress, the plants exhibit a ‘water-saving effect’ by closing their stomata, thereby lowering gsw [25,33]. This is usually mediated by the increased accumulation of abscisic acid (ABA) in the guard cells [34]. The observed decline in gsw due to drought stress under both CO_2_ conditions reduced the transpiration rate. Stomatal closure reduces CO_2_ concentration in mesophyll cells, increasing the CO_2_ concentration gradient for diffusion and thereby increasing water use efficiency [35]. This is attributed to the observed reduction in the Ci/Ca ratio and the increased WUEi under aCO_2_ + drought conditions compared to aCO_2_ + control conditions.

In contrast, under eCO_2_, drought-affected plants exhibit a ‘low Ci effect’ to cope with adverse effects on plant carbon fixation [25,26]. The study observed that under eCO_2_ + drought conditions, Ci/Ca, WUEi, and WUEinst are on par with those under the eCO_2_ + control condition, indicating sufficient carbon availability for photosynthesis. It has been reported that the drought-induced reduction in Ci modifies photosynthetic activity during the steep initial linear phase of the photosynthetic–CO_2_ response curve [36]. Hence, the relative response of photosynthesis to eCO_2_ becomes more pronounced under drought conditions [25,26]. Under eCO_2_ + drought conditions, the availability of carbon sources did not improve WUEinst, suggesting non-stomatal limitation of photosynthesis in addition to reduced gsw, including decreased mesophyll conductance [37] and biochemical limitations, such as temporary or permanent inactivation of Rubisco [38]. Drought-induced non-stomatal limitations, such as reduced RuBP and ATP contents, have been reported in C_3_ plants [39]. In addition, drought induces downregulation of photosystem II, affecting ATP and NADPH production through the electron transport chain [40]. Both stomatal and non-stomatal changes depend on changes in soil-water status [41]. It has also been reported that non-stomatal limitations increase with increasing drought stress intensity [42]. A meta-analysis of eCO_2_ and drought stress revealed that WUEi increased in proportion to eCO_2_, whereas plant WUE was less responsive [43]. This was evident from the observed non-significant effect of CO_2_ on midday leaf water potential, despite a reduction under drought stress. Overall, drought-mediated negative feedback, such as hydraulic and chemical signaling, decreases CO_2_ availability in chloroplasts, thereby lowering the photosynthetic rate [44]. In contrast, eCO_2_ conditions mitigate the adverse effects of drought.

The drought-induced reduction in photosynthesis results in carbon limitation for plant growth. The observed 25% reduction in plant height and 15–17% reduction in the number of mainstem nodes under drought conditions indicate the biomass limitations under water-deficit stress. This is particularly due to reduced photosynthesis resulting from stomatal or non-stomatal limitations and impaired transport of photo-assimilates [45,46]. It has been reported that drought stress impairs seedling growth, primarily due to disrupted cell elongation and reduced cell division resulting from an interrupted water supply to actively growing meristematic cells [47]. Several studies have reported significant declines in the number of leaves and tillers, leaf surface area, internode elongation, shoot length, and fresh and dry matter under drought stress [47,48]. The total biomass of plants grown under drought conditions declined by 23% and 24% under aCO_2_ and eCO_2_, respectively, compared to the control. Drought stress reduces leaf surface area and thus the active area for photosynthesis, leading to shedding of plant organs such as leaves, squares, and bolls [49]. The proportion of the reproductive part was reduced under drought stress relative to well-watered conditions, and eCO_2_ conditions did not affect it. Conversely, the plants diverted relatively more biomass to the root system in response to drought stress, thereby increasing water absorption from the deeper soil layer, a phenomenon termed hydrotropism [50]. This selective allocation of biomass to the root system rather than reproductive structures will be a drought-adaptive adjustment of the plant, enabling increased water absorption. Similar observations have been reported in multiple crops under drought stress [51,52,53]. This response reflects a drought-avoidance mechanism that plants have evolved to prevent dehydration during transient periods of drought stress [54].

Plants exposed to drought stress produced fewer bolls per day, resulting in a lower total number of bolls per plant. Although plants grown under eCO_2_ conditions increased the number of bolls under control and drought conditions, the increase was not significant. In cotton, the first square-to-first flower and first flower-to-peak bloom stages are sensitive to abiotic stresses, especially drought [30]. It affects the development of fruiting sites and induces abortion of existing fruits, thereby reducing yield [55]. It has been reported that cotton plants shed more squares than bolls under drought conditions, resulting in fewer bolls [56]. In this study, boll retention was not adversely affected by drought stress under either aCO_2_ or eCO_2_ conditions, suggesting that the reduced boll number observed under drought was likely due to increased square abscission rather than boll loss. Drought-induced oxidative stress during the reproductive stage suppresses carbohydrate metabolism and reduces assimilation in reproductive parts, leading to square and flower shedding [57]. Studies reported that drought stress enhanced the hydrolysis of cellulose and pectin at the base of the pedicel, resulting in significant degradation of epidermal, cortical, and phloem cells, leading to the formation of an abscission zone and causing a square drop [58]. The boll maturation period was shortened under drought, and eCO_2_ accelerated this process under stress. Generally, plants accelerate their maturation in response to drought to avoid unfavorable conditions [59]. However, under eCO_2_, enhanced water-use efficiency and greater availability of assimilates may have provided the necessary resources for boll development. As a result, the boll filling process occurred more rapidly, leading to a shorter maturation period even under drought stress.

In this study, drought stress and eCO_2_ did not significantly affect pollen grain production, in contrast to previous reports indicating that drought stress negatively affects anther development and pollen viability [60]. This may be due to the cultivar’s genetic tolerance. On the other hand, drought stress significantly reduced the number of ovules per flower. The observed limitation in photo-assimilates, along with reduced translocation to reproductive organs, contributed to decreased ovule production under drought stress [61]. In addition, drought stress damaged the cytological structure of developing ovules and increased reactive oxygen species accumulation in the pistil during ovule development [62]. It has been reported that drought stress limits soluble starch synthase and ADP-glucose pyrophosphorylase activities [63] and downregulates the activities of genes controlling sucrose transport, sucrose synthase, and invertase activities in the pistil [63,64]. This subsequently impairs pollen tube growth, affecting successful fertilization [63]. It is important to note that microsporogenesis in cotton begins earlier in floral development than the differentiation of ovule integuments and nucellus [65]. Since drought was imposed at the flowering stage, pollen number, determined during earlier developmental phases, may have been established before the onset of stress effects on plants. In contrast, ovule development, which continues later, remained susceptible to drought stress, thereby affecting its production. In addition, under assimilative limitations imposed by drought, preferential allocation of resources towards developing pollen rather than ovules, which require a sustained supply of assimilates during and after fertilization, may have further contributed to the differential response of the two reproductive structures. Hence, this study suggests that ovule abortion is the dominant factor responsible for yield reduction in cotton.

As a consequence of the adverse effect of drought stress on gas exchange and reproductive growth, both seed and lint yield in cotton were significantly reduced [66]. Reduced biomass allocation to reproductive structures has likely contributed to the lower seed cotton and lint yield observed under drought stress. Since eCO_2_ increased the reproductive biomass proportion by only 3% relative to drought alone (drought + aCO_2_: 34. 2% and drought_eCO_2_: 37.6%), the corresponding gain in lint weight under drought + eCO_2_ was modest (4%), and seed weight showed no comparable increase. Furthermore, the BMP was shortened under drought at eCO_2_, leaving plants less time to allocate biomass to reproductive structures. This study therefore highlights that CO_2_ fertilization can only partially alleviate the adverse effects of drought. The plants responded positively to eCO_2_, with increases in seed cotton and lint weights. Reduced availability of photo-assimilates and the number of ovules under drought stress resulted in an 18% decline in seed cotton weight and a 17% decline in lint weight compared to the control. The proportion of lint did not change due to either drought or eCO_2_. Multiple experiments reported similar findings [67,68]. Both seed and lint weights were significantly affected by drought stress, resulting in no significant change in the proportion of lint. In addition to reducing fiber yield, drought stress adversely affected fiber quality, resulting in increased micronaire and reduced fiber length. The process of fiber development comprises four stages: fiber initiation through differentiation from the protoderm, fiber cell elongation, secondary cell wall deposition, and maturation [69]. Among the four, the first three stages are sensitive to drought stress because they require maintaining cell turgor and carbohydrate assimilation for fiber development [70]. The downregulation of the sucrose synthesis gene involved in seed fiber development has been reported under drought stress, resulting in shorter fibers [70]. In addition, osmotic stress during the fiber initiation and elongation stages reduces fiber cell division, resulting in fewer fiber cells [60]. It has also been reported that photosynthate allocation to fiber and embryos was reduced under drought conditions, as a strategy to maintain the seed coat and protect the embryo against adverse environmental conditions [71]. A transcriptomic study indicated that while drought stress has minimal effect on fiber initiation, it significantly disrupts fiber elongation by downregulating essential genes involved in cell wall loosening and expansion [72]. However, eCO_2_ did not influence the development and maturation of cotton fibers.

Overall, CO_2_ fertilization helps partially mitigate drought-induced yield loss through several interconnected processes. During drought conditions, stomata close, which restricts the diffusion of CO_2_. This, along with non-stomatal limitations, reduces carbon assimilation and the supply of photoassimilates to the plant’s reproductive parts. eCO_2_ directly influences carbon assimilation: by increasing intercellular CO_2_ concentration, it maintains carboxylation even at low stomatal conductance, thereby keeping the ratio of internal to external CO_2_ concentrations (Ci/Ca) and water use efficiency (WUEi) under drought conditions comparable to those in well-watered controls. This greater availability of assimilates helps offset the reduction in reproductive biomass filling and supports a slight recovery in the proportion of reproductive biomass (from 34.2% to 37.6%) and lint weight. Since the number of ovules relies on the supply of assimilates to the pistil during a critical period, the additional carbon under eCO_2_ likely reduced ovule abortion and partially preserved the reproductive sink. However, the compensation was incomplete; eCO_2_ did not restore ovule number or seed weight to control levels, and faster boll maturation shortened the filling period. Thus, while eCO_2_ alleviates the carbon limitations that impair reproductive development, the mitigation remains partial because non-stomatal limitations persist, and the maturation window is reduced.

## 4. Materials and Methods

### 4.1. Experimental Facility and Setup

The experiment was conducted in the Soil–Plant–Atmospheric Research (SPAR) facility at the Environmental Plant Physiology Laboratory, Mississippi State University, Mississippi State, MS, USA, during the summer of 2023 (Figure S2). The SPAR units contain a steel soil bin and a transparent Plexiglas chamber, designed to house the plant canopy. The Plexiglas chambers are 1.27 cm thick and allow 97% of solar radiation to pass [73]. These chambers accurately monitor and maintain the required temperature and CO_2_ through an automated control system, with temperature regulated by integrating heating and cooling units. The chambers were sealed to maintain the gas flow, with a door for easy access for measurements. Since the chambers were in open sunlight, plants were grown under natural sunlight conditions. The details and specifications of the SPAR units have been presented by Reddy et al. [74].

An upland cotton cultivar, DP 1646 B2XF (Deltapine, Bayer Crop Science, St. Louis, MO, USA) , was grown in pots (15 cm diameter × 65 cm height) filled with a 3:1 volume ratio of fine sand and topsoil mix (87% sand, 2% clay, and 11% silt). The bottom of the pot contains a 1-cm-diameter hole and 250 g of gravel for easy drainage. Twelve plants were grown in each SPAR unit as replications. Thus, 48 pots were arranged in four SPAR units. Four seeds were sown in each pot, and the seedlings were thinned to one after the fourth leaf stage. The plants grew under outdoor conditions up to flowering, and water and nutrients were supplied via Hoagland’s nutrient solution [75] three times a day.

### 4.2. Treatments

The drought and CO_2_ treatments were imposed during the flowering stage. Plants were initially grown outdoors under natural environmental conditions and, at the onset of flowering (60 days after planting), the plants were transferred to four SPAR units (12 pots per unit) and preconditioned at two CO_2_ concentrations: 425 ppm (ambient, aCO_2_) and 725 ppm (enriched, eCO_2_). Two units were assigned to each concentration. Two levels of drought, control (well-watered, 0.12 m^3^ H_2_O m^−3^ volumetric soil moisture content) and drought (0.09 m^3^ H_2_O m^−3^ volumetric soil moisture content), were applied under both aCO_2_ and eCO_2_ conditions. Thus, the experiment contains combinations of drought and CO_2_ levels. The CO_2_ levels in each SPAR unit are continuously monitored and adjusted every 10 s throughout the day [62]. During daylight hours, CO_2_ levels were maintained within 10 ppm of the target set point by adjusting the controls. A mass-balance approach, using the output from dedicated CO_2_ analyzers for each unit (Model LI 6200, LI-COR Inc., Lincoln, NE, USA), was employed to control the solenoid valves as required. To regulate CO_2_ in each chamber, pure CO_2_ is injected via a system comprising a pressure regulator, a solenoid, needle valves, and a flowmeter. These flowmeters are calibrated with a gas displacement meter at the start and end of each experiment [74]. The treatments were continued until the harvest. Physiology, boll production, yield, and fiber quality parameters were recorded during the study. The soil moisture content in each treatment was monitored using Decagon soil moisture sensor probes (ECH_2_O, EC-5, Decagon Devices, Inc., Pullman, WA, USA) inserted to a depth of 15 cm in four randomly selected pots. All SPAR units were maintained at 30/22 °C (day/night) throughout the experiment. The experiment used a two-factorial, completely randomized design with CO_2_ and drought as factors. Black mesh shade cloths were placed along the edges of the Plexiglas chambers to mimic the effect of surrounding border plants. The shade net was adjusted periodically to accommodate plant growth.

### 4.3. Measurements

#### 4.3.1. Midday Leaf Water Potential

Uppermost fully expanded leaves were collected from five randomly selected plants per treatment between 12.00 and 13.00 h during the boll development stage. The midday leaf water potential was measured immediately after sampling using a pressure chamber (Soil Moisture Equipment Corp., Santa Barbara, CA, USA) [23].

#### 4.3.2. Gas Exchange Parameters

The plant physiology measurements were recorded on the uppermost fully matured leaf (5th leaf from the top) during the boll development stage. The gas exchange measurements were recorded using a photosynthesis system (LI-6400, LI-COR Environmental, Lincoln, NE, USA) between 11.00 and 13:00 h. All the measurements were collected from five plants per treatment. The conditions for leaf stabilization were a leaf temperature of 30 °C, 40–50% relative humidity, a CO_2_ concentration of 425 or 725 µmol mol^−1^ depending on the treatment, 1500 µmol m^−2^ s^−1^ photosynthetically active radiation, and a flow rate of 500 µmol s^−1^. The gas exchange measurements, including photosynthesis rate (µmol CO_2_ m^−2^ s^−1^), stomatal conductance (gsw; mol H_2_O m^−2^ s^−1^), and transpiration rate (mmol H_2_O m^−2^ s^−1^), were measured simultaneously. The Ci/Ca (leaf internal CO_2_ concentration/ambient CO_2_ concentration) was obtained from the photosynthetic system. The intrinsic water use efficiency (WUEi; µmol CO_2_ (mol H_2_O)^−1^) and instantaneous water use efficiency (WUEinst; µmol CO_2_ (mmol H_2_O)^−1^) were compared using the following formula.

$$WUEi=\frac{Photosynthesis\;rate\;(µmol\;{CO}_{2}\;m^{-2}\;s^{-1})}{Stomatal\;conductance\;(mol\;H_{2}O\;m^{2}\;s^{-1})}$$

$$WUEinst=\frac{Photosynthesis\;rate\;(µmol\;{CO}_{2}\;m^{-2}\;s^{-1})}{Transipration\;rate\;(mmol\;H_{2}O\;m^{2}\;s^{-1})}$$

#### 4.3.3. Plant Growth and Biomass

Plant growth measurements, such as plant height and number of mainstem nodes, were recorded on the day of harvest, 135 days after sowing. Plant height (cm) was measured from the cotyledonary node to the stem apex. The number of mainstem nodes was counted per plant, excluding the cotyledonary node, up to the uppermost fully expanded leaf. After harvest, plants were separated into stems, leaves, bolls, and roots. Each component was placed in a brown paper bag and oven-dried at 80 °C for 72 h to record dry biomass (g).

#### 4.3.4. Boll Production and Retention

The bolls were tagged daily at anthesis throughout the experiment, for about 70 consecutive days from the treatment. Each tag was labeled with the flowering date to track the number of days. During the process, the abscised bolls were collected and recorded. Thus, the number of open and retained bolls was recorded daily across treatments. Towards the end of the experiment, the bolls were observed for dehiscence, indicated by boll cracking and visible lint. This data was used to determine the boll maturation period (BMP), defined as the time from anthesis to boll dehiscence. The total number of bolls produced and retained was counted at harvest (135 days after sowing), and boll retention (%) was calculated as the number of bolls retained divided by the total number of bolls produced per plant.

#### 4.3.5. Pollen and Ovule Production

The number of pollen and ovules was counted in three randomly selected flowers collected between 18.00 and 19.00 h before anthesis. The pollen grains were counted by cutting the indehiscent anther with a needle in a drop of water using a Stereo microscope (Nikon SMZ800, Fujisawa, Japan) at 10× magnification. Three anthers were randomly selected from each replicate across all treatments. The number of ovules was counted by longitudinally cutting the ovary.

Pollen grains were collected shortly after anthesis, between 18:00 and 19:00 h, for morphological analysis. Anthers from four individual plants per treatment were gently tapped over Petri dishes to dislodge the pollen. The collected pollen was placed on a steel stub and sputter-coated with platinum using an EMS150T ES coater (Quorum Technologies, Electron Microscopy Sciences, Morgantown, PA, USA). Morphological examination was carried out using a scanning electron microscope (JEOL JSM-6500F, JEOL USA, Inc., Peabody, MA, USA) operated at 5 kV, and images were captured at magnifications of 400× and 2000×.

#### 4.3.6. Seed and Lint Yield

The opened bolls were collected at harvest, and the seed cotton weight (g), lint weight (g), and lint proportion (%) were recorded for each plant cross treatment. Lint yield was determined after ginning the seed cotton using a roller gin. The lint from all 12 plants in each treatment was bulked to obtain samples for fiber quality testing. Three subsamples of 10 g each were drawn from the bulk and subjected to fiber quality testing using High Volume Instrumentation (HVI) by the Fiber and Biopolymer Research Institute at Texas Tech University, Lubbock, TX, USA, as described in [76]. The analysis provides fiber length (mm), fiber strength (g tex^−1^), micronaire (unitless), and uniformity (%).

### 4.4. Data Analysis

A two-way factorial completely randomized design was used to perform the analysis of variance (ANOVA) in R (version 4.1.1), utilizing the doebioresearch package [77]. Post hoc comparisons were carried out using the LSD test at a 5% significance level to determine differences among treatments. Visualizations of the results were created using SigmaPlot version 13.0 (Systat Software, Inc., San Jose, CA, USA) and GraphPad Prism version 8.00 (GraphPad Software, San Diego, CA, USA).

## 5. Conclusions

This study highlights that CO_2_ fertilization is effective under well-watered and drought conditions, and that the detrimental impacts of drought stress on cotton growth, reproductive physiology, yield, and fiber quality were partially mitigated under eCO_2_. Drought-induced declines in gas exchange parameters, biomass accumulation, and reproductive traits, such as ovule production and boll development, led to significant reductions in both seed and lint yields. While drought stress impaired photosynthetic activity and water-use efficiency under control conditions, eCO_2_ enhanced gas exchange, thereby increasing WUE. Although pollen viability remained unaffected, drought-induced abortion of squares and ovules significantly constrained yield potential. The reduction in fiber quality under drought, evidenced by increased micronaire and shorter fiber length, was linked to disrupted sucrose metabolism and impaired cell elongation. Overall, the finding emphasizes the detrimental effects of drought stress and the partial mitigation of eCO_2_ on gas exchange, WUE, and yield. However, since the study was conducted in a controlled environment with a single cultivar, the findings have limited broader applicability. Future research should examine the plants under natural field conditions to improve field applications.

## Figures and Tables

**Table 1:** Analysis of variance of the effects of enriched CO_2_, drought stress, and their combination on morpho-physiological and yield-related traits in cotton.

Parameter	CO_2_	Drought	CO_2_ × Drought
Photosynthesis (µmol CO_2_ m^−2^ s^−1^)	***	***	ns
Stomatal conductance (mol H_2_O m^−2^ s^−1^)	**	***	**
Transpiration (mmol H_2_O m^−2^ s^−1^)	ns	***	***
Ci/Ca	ns	**	ns
Intrinsic water use efficiency (WUEi)	**	*	ns
Instantaneous water use efficiency (WUEinst)	**	*	ns
Plant height (cm)	ns	***	ns
Mainstem Nodes (no. plant^−1^)	ns	***	*
Stem biomass (g plant^−1^)	ns	***	ns
Root biomass (g plant^−1^)	ns	**	ns
Green Boll biomass (g plant^−1^)	ns	***	ns
Square biomass (g plant^−1^)	ns	***	ns
Seed Cotton weight (g plant^−1^)	**	**	ns
Lint weight (g plant^−1^)	***	***	ns
Seed yield (g plant^−1^)	**	***	ns
Lint (%)	ns	ns	ns
Boll maturation period (days)	*	***	ns
Ovules (no. flower^−1^)	ns	***	ns
Pollens (no. anther^−1^)	ns	ns	ns
Micronaire (unitless)	ns	***	ns
Fiber length (mm)	ns	***	ns
Uniformity (%)	ns	ns	ns
Fiber strength (g tex^−1^)	ns	ns	ns

*, **, and *** represent statistical significance at *p* < 0.05, 0.01, and 0.001, respectively. ‘ns’ indicates non-significance.

**Table 2:** Open bolls, boll maturation period, and boll retention of cotton under control and drought conditions at ambient (aCO_2_; 425 ppm) and enriched (eCO_2_; 725 ppm) CO_2_.

CO_2_ Level	Soil Moisture Status	Open Bolls (No. Plant^−1^)	Boll Maturation Period (Days)	Boll Retention (%)
aCO_2_	Control	24.9 a *	47.3 a	92.9 b
aCO_2_	Drought	17.2 b	45.9 b	97.9 a
eCO_2_	Control	27.0 a	47.1 a	85.8 c
eCO_2_	Drought	19.2 b	44.7 c	85.5 c

* The letter beside the values indicates significance at *p* < 0.05. Treatments with the same letters are not significantly different.

## Data Availability

The original contributions presented in this study are included in the article/Supplementary Materials. Further inquiries can be directed to the corresponding authors.

## Supplementary Materials

The following supporting information can be downloaded at https://www.mdpi.com/article/10.3390/plants15142189/s1. Figure S1: Cotton plants under control (well-watered, 0.12 m^3^ H_2_O m^−3^ volumetric soil moisture content) and drought (0.09 m^3^ H_2_O m^−3^ volumetric soil moisture content) conditions under two CO_2_ concentrations—425 ppm (ambient, aCO_2_) and 725 ppm (enriched, eCO_2_)—in the Soil–Plant–Atmospheric-Research facility. Figure S2: The Soil–Plant–Atmosphere-Research facility (SPAR) used in the study located at Environmental Plant Physiology Laboratory, Mississippi State University, MS, USA. Each SPAR facility consists of a plexiglass chamber to hold the plant canopy and metal bin to accommodate the root system. A heating and cooling system is present at the rear side of the facility which controls the temperature inside the chamber. The CO_2_ levels are monitored by a CO_2_ analyzer and adjusted through the control system.

## References

[1] Brodersen C.R., Roddy A.B., Wason J.W., McElrone A.J. (2019). Functional status of xylem through time. Annu. Rev. Plant Biol..

[2] Iqbal S., Wang X., Mubeen I., Kamran M., Kanwal I., Díaz G.A., Abbas A., Parveen A., Atiq M.N., Alshaya H. (2021). Phytohormones trigger drought tolerance in crop plants: Outlook and future perspectives. Front. Plant Sci..

[3] Eck M.A., Murray A.R., Ward A.R., Konrad C.E. (2020). Influence of growing season temperature and precipitation anomalies on crop yield in the southeastern United States. Agric. For. Meteorol..

[4] NOAA-NIDIS Drought Basics. https://www.drought.gov/what-is-drought/drought-basics.

[5] Mishra A.K., Singh V.P. (2010). A review of drought concepts. J. Hydrol..

[6] USDA Cotton Outlook: Cotton Production and Abandonment Rates. https://www.usda.gov/sites/default/files/documents/2025AOF-cotton-outlook.pdf.

[7] Carver J.L. (2022). Texas’s Cotton Industry is Facing Its Worst Harvest in Years, Costing the State More Than $2 Billion. The Texas Tribune.

[8] Constable G.A., Bange M.P. (2015). The yield potential of cotton (*Gossypium hirsutum* L.). Field Crops Res..

[9] Dorman S.J., Taylor S.V., Malone S., Roberts P.M., Greene J.K., Reisig D.D. (2022). Sampling optimization and crop interface effects on *Lygus lineolaris* populations in southeastern USA cotton. Insects.

[10] Marfo J.N., Ambinakudige S., Beegum S., Reddy K.R. (2026). The impact of temperature and water stress on cotton planting windows and fiber quality in Mississippi. Field Crops Res..

[11] Gao M., Xu B., Wang Y., Zhou Z., Hu W. (2021). Quantifying individual and interactive effects of elevated temperature and drought stress on cotton yield and fibre quality. J. Agron. Crop Sci..

[12] Liu S., Zhang W., Shi T., Li T., Li H., Zhou G., Wang Z., Ma X. (2025). Increasing exposure of cotton growing areas to compound drought and heat events in a warming climate. Agric. Water Manag..

[13] Zahra N., Hafeez M.B., Kausar A., Al Zeidi M., Asekova S., Siddique K.H.M., Farooq M. (2023). Plant photosynthetic responses under drought stress: Effects and management. J. Agron. Crop Sci..

[14] Sun L., Wang Y., Zhang J. (2026). Calcium signaling in plant defense. New Plant Prot..

[15] Qiao M., Hong C., Jiao Y., Hou S., Gao H. (2024). Impacts of drought on photosynthesis in major food crops and the related mechanisms of plant responses to drought. Plants.

[16] Fujita M., Hasanuzzaman M. (2022). Approaches to enhancing antioxidant defense in plants. Antioxidants.

[17] Li X., Zhao Y., Gao C., Li X., Wu K., Lin M., Sun W. (2025). Integrated analysis of physiological responses and transcriptome of cotton seedlings under drought stress. Int. J. Mol. Sci..

[18] Sheri V., Kumar M., Jaconis S., Zhang B. (2023). Antioxidant defense in cotton under environmental stresses: Unraveling the crucial role of a universal defense regulator for enhanced cotton sustainability. Plant Physiol. Biochem..

[19] Saeed M., Naeem M., Javed A., Perveen S., Sajjad I., Yousaf M.Z., Chohan M.S.M., Riaz M., Ullah S., Song X. (2024). Characterization of water deficit tolerance in upland cotton (*Gossypium hirsutum* L.) assessing morphological, biochemical, molecular and yield attributes. Acta Physiol. Plant..

[20] Fathi A., Shiade S.R.G., Saleem A., Shohani F., Fazeli A., Riaz A., Zulfiqar U., Shabaan M., Ahmed I., Rahimi M. (2025). Reactive oxygen species (ROS) and antioxidant systems in enhancing plant resilience against abiotic stress. Int. J. Agron..

[21] Witt T.W., Habimana A., Sanchez J., Ulloa M. (2024). Assessing agronomic and physiological traits during reproductive developmental stages for breeding upland drought-tolerant cotton. Agrosyst. Geosci. Environ..

[22] Sun F., Chen Q., Chen Q., Jiang M., Qu Y. (2023). Yield-based drought tolerance index evaluates the drought tolerance of cotton germplasm lines in the interaction of genotype-by-environment. PeerJ.

[23] Lokhande S., Reddy K.R. (2014). Reproductive and fiber quality responses of upland cotton to drought stress. Agron. J..

[24] Zhang J., Loka D.A., Wang J., Ran Y., Shao C., Tuersun G., Li Y., Wang S., Zhou Z., Hu W. (2024). Co-occurring elevated temperature and drought stress inhibit cotton pollen fertility by disturbing anther carbohydrate and energy metabolism. Ind. Crops Prod..

[25] Duursma R.A., Medlyn B.E. (2012). MAESPA: A model to study interactions between water limitation, environmental drivers and vegetation function at tree and stand levels, with an example application to [CO_2_] × drought interactions. Geosci. Model Dev..

[26] Kelly J.W.G., Duursma R.A., Atwell B.J., Tissue D.T., Medlyn B.E. (2016). Drought × CO_2_ interactions in trees: A test of the low-intercellular CO_2_ concentration (Ci) mechanism. New Phytol..

[27] Hao L., Chang Z., Lu Y., Tian Y., Zhou H., Wang Y., Wu J. (2023). Drought dampens the positive acclimation responses of leaf photosynthesis to elevated [CO_2_] by altering stomatal traits, leaf anatomy, and Rubisco gene expression in Pyrus. Environ. Exp. Bot..

[28] Broughton K.J., Smith R.A., Duursma R.A., Tan D.K., Payton P., Bange M.P., Tissue D.T. (2017). Warming alters the positive impact of elevated CO_2_ concentration on cotton growth and physiology during soil water deficit. Funct. Plant Biol..

[29] Bista M.K., Ramamoorthy P., Vennam R.R., Poudel S., Reddy K.R., Bheemanahalli R. (2025). Impacts of abiotic stresses on cotton physiology and vigor under current and future CO_2_ levels. J. Integr. Agric..

[30] Ul-Allah S., Rehman A., Hussain M., Farooq M. (2021). Fiber yield and quality in cotton under drought: Effects and management. Agric. Water Manag..

[31] Martínez-Vilalta J., Garcia-Forner N. (2017). Water potential regulation, stomatal behaviour and hydraulic transport under drought: Deconstructing the iso/anisohydric concept. Plant Cell Environ..

[32] Choat B., Jansen S., Brodribb T.J., Cochard H., Delzon S., Bhaskar R., Bucci S.J., Feild T.S., Gleason S.M., Hacke U.G. (2012). Global convergence in the vulnerability of forests to drought. Nature.

[33] Yang Y.-J., Bi M.-H., Nie Z.-F., Jiang H., Liu X.-D., Fang X.-W., Brodribb T.J. (2021). Evolution of stomatal closure to optimize water-use efficiency in response to dehydration in ferns and seed plants. New Phytol..

[34] Assmann S.M., Jegla T. (2016). Guard cell sensory systems: Recent insights on stomatal responses to light, abscisic acid, and CO_2_. Curr. Opin. Plant Biol..

[35] Medrano H., Tomás M., Martorell S., Flexas J., Hernández E., Rosselló J., Pou A., Escalona J.-M., Bota J. (2015). From leaf to whole-plant water use efficiency (WUE) in complex canopies: Limitations of leaf WUE as a selection target. Crop J..

[36] Ellsworth D.S., Thomas R., Crous K.Y., Palmroth S., Ward E., Maier C., DeLucia E., Oren R. (2012). Elevated CO_2_ affects photosynthetic responses in canopy pine and subcanopy deciduous trees over 10 years: A synthesis from Duke FACE. Glob. Change Biol..

[37] Diaz-Espejo A., Nicolás E., Fernández J.E. (2007). Seasonal evolution of diffusional limitations and photosynthetic capacity in olive under drought. Plant Cell Environ..

[38] Feller U. (2016). Drought stress and carbon assimilation in a warming climate: Reversible and irreversible impacts. J. Plant Physiol..

[39] Flexas J., Medrano H. (2002). Drought-inhibition of photosynthesis in C_3_ plants: Stomatal and non-stomatal limitations revisited. Ann. Bot..

[40] Saha S., Johnson G.N. (2025). Divergent effects of successive drought and flooding on photosynthesis in wheat and barley. Front. Plant Sci..

[41] Drake J.E., Power S.A., Duursma R.A., Medlyn B.E., Aspinwall M.J., Choat B., Creek D., Eamus D., Maier C., Pfautsch S. (2017). Stomatal and non-stomatal limitations of photosynthesis for four tree species under drought: A comparison of model formulations. Agric. For. Meteorol..

[42] Salmon Y., Lintunen A., Dayet A., Chan T., Dewar R., Vesala T., Hölttä T. (2020). Leaf carbon and water status control stomatal and nonstomatal limitations of photosynthesis in trees. New Phytol..

[43] Wang Z., Wang C., Liu S. (2022). Elevated CO_2_ alleviates adverse effects of drought on plant water relations and photosynthesis: A global meta-analysis. J. Ecol..

[44] Buckley T.N. (2019). How do stomata respond to water status?. New Phytol..

[45] Zargar S.M., Gupta N., Nazir M., Mahajan R., Malik F.A., Sofi N.R., Shikari A.B., Salgotra R.K. (2017). Impact of drought on photosynthesis: Molecular perspective. Plant Gene.

[46] Das S., Rawat P., Shankhdhar D., Shankhdhar S.C. (2020). Drought stress: An impact of climate change, its consequences and amelioration through silicon (Si). Sustainable Agriculture in the Era of Climate Change.

[47] Zia R., Nawaz M.S., Siddique M.J., Hakim S., Imran A. (2021). Plant survival under drought stress: Implications, adaptive responses, and integrated rhizosphere management strategy for stress mitigation. Microbiol. Res..

[48] Fahad S., Bajwa A.A., Nazir U., Anjum S.A., Farooq A., Zohaib A., Sadia S., Nasim W., Adkins S., Saud S. (2017). Crop production under drought and heat stress: Plant responses and management options. Front. Plant Sci..

[49] Ergashovisch K.A., Azamatovna A.Z., Toshtemirovna N.U., Rakhimovna A.K. (2020). Ecophysiological effects of water deficiency on cotton varieties. J. Crit. Rev..

[50] Bodner G., Nakhforoosh A., Kaul H.-P. (2015). Management of crop water under drought: A review. Agron. Sustain. Dev..

[51] Fang Y., Xiong L. (2015). General mechanisms of drought response and their application in drought resistance improvement in plants. Cell. Mol. Life Sci..

[52] Walne C.H., Thenveettil N., Ramamoorthy P., Bheemanahalli R., Reddy K.N., Reddy K.R. (2024). Unveiling drought-tolerant corn hybrids for early-season drought resilience using morpho-physiological traits. Agriculture.

[53] Abdullah M.M., Waraich E.A., Ahmad M., Hussain S., Asghar H.N., Haider A., Zulfiqar U., Ahmad Z., Soufan W., Prasad P.V. (2025). Improving soybean drought tolerance via silicon-induced changes in growth, physiological, biochemical, and root characteristics. Plant Signal. Behav..

[54] Kooyers N.J. (2015). The evolution of drought escape and avoidance in natural herbaceous populations. Plant Sci..

[55] Zonta J.H., Brandão Z.N., Rodrigues J.I.D.S., Sofiatti V. (2017). Cotton response to water deficits at different growth stages. Rev. Caatinga.

[56] Chen H., Feng H., Zhang X., Zhang C., Wang T., Dong J. (2019). An *Arabidopsis* E3 ligase HUB2 increases histone H2B monoubiquitination and enhances drought tolerance in transgenic cotton. Plant Biotechnol. J..

[57] Loka D.A., Oosterhuis D.M., Baxevanos D., Vlachostergios D., Hu W. (2019). How potassium deficiency alters flower bud retention on cotton (*Gossypium hirsutum* L.). Arch. Agron. Soil Sci..

[58] Yu H., Luo Y., Cao N., Wang S., Zhou Z., Hu W. (2024). Drought-induced cell wall degradation in the base of pedicel is associated with accelerated cotton square shedding. Plant Physiol. Biochem..

[59] Shavrukov Y., Kurishbayev A., Jatayev S., Shvidchenko V., Zotova L., Koekemoer F., de Groot S., Soole K., Langridge P. (2017). Early flowering as a drought escape mechanism in plants: How can it aid wheat production?. Front. Plant Sci..

[60] Zhang M., Zhang X., Guo L., Qi T., Liu G., Feng J., Shahzad K., Zhang B., Li X., Wang H. (2020). Single-base resolution methylome of cotton cytoplasmic male sterility system reveals epigenomic changes in response to high-temperature stress during anther development. J. Exp. Bot..

[61] Glenny W.R., Runyon J.B., Burkle L.A. (2018). Drought and increased CO_2_ alter floral visual and olfactory traits with context-dependent effects on pollinator visitation. New Phytol..

[62] Cheng M., Wang Z., Cao Y., Zhang J., Yu H., Wang S., Zhou Z., Hu W. (2024). Soil drought during the development of cotton ovule destroyed the antioxidant balance of cotton pistil to hinder the ovule formation. J. Agron. Crop Sci..

[63] Hu W., Liu Y., Loka D.A., Zahoor R., Wang S., Zhou Z. (2019). Drought limits pollen tube growth rate by altering carbohydrate metabolism in cotton (*Gossypium hirsutum*) pistils. Plant Sci..

[64] Hu W., Huang Y., Bai H., Liu Y., Wang S., Zhou Z. (2020). Influence of drought stress on pistil physiology and reproductive success of two *Gossypium hirsutum* cultivars differing in drought tolerance. Physiol. Plant..

[65] de Moura S.M., Rossi M.L., Artico S., Grossi-de-Sa M.F., Martinelli A.P., Alves-Ferreira M. (2020). Characterization of floral morphoanatomy and identification of marker genes preferentially expressed during specific stages of cotton flower development. Planta.

[66] Gören H.K., Tan U. (2024). Unraveling the complexities of drought stress in cotton: A multifaceted analysis of selection criteria and breeding approaches. PeerJ.

[67] Niu J., Zhang S., Liu S., Ma H., Chen J., Shen Q., Ge C., Zhang X., Pang C., Zhao X. (2018). The compensation effects of physiology and yield in cotton after drought stress. J. Plant Physiol..

[68] Dietz K.-J., Zörb C., Geilfus C.-M. (2021). Drought and crop yield. Plant Biol..

[69] Rehman A., Farooq M. (2019). Morphology, physiology and ecology of cotton. Cotton Production.

[70] Gao M., Snider J.L., Bai H., Hu W., Wang R., Meng Y., Wang Y., Chen B., Zhou Z. (2020). Drought effects on cotton (*Gossypium hirsutum* L.) fibre quality and fibre sucrose metabolism during the flowering and boll-formation period. J. Agron. Crop Sci..

[71] Zhu H., Hu W., Li Y., Zou J., He J., Wang Y., Meng Y., Chen B., Zhao W., Wang S. (2024). Drought decreases cotton fiber strength by altering sucrose flow route. J. Exp. Bot..

[72] Padmalatha K.V., Dhandapani G., Kanakachari M., Kumar S., Dass A., Patil D.P., Rajamani V., Kumar K., Pathak R., Rawat B. (2012). Genome-wide transcriptomic analysis of cotton under drought stress reveal significant down-regulation of genes and pathways involved in fibre elongation and up-regulation of defense responsive genes. Plant Mol. Biol..

[73] Zhao D., Reddy K.R., Kakani V.G., Read J.J., Sullivan J.H. (2003). Growth and physiological responses of cotton (*Gossypium hirsutum* L.) to elevated carbon dioxide and ultraviolet-B radiation under controlled environmental conditions. Plant Cell Environ..

[74] Reddy K.R., Hodges H.F., Read J.J., McKinion J.M., Baker J.T., Tarpley L., Reddy V.R. (2001). Soil-Plant-Atmosphere-Research (SPAR) facility: A tool for plant research and modeling. Biotronics.

[75] Hewitt E.J. (1953). Sand and water culture methods used in the study of plant nutrition. Soil Sci. Soc. Am. J..

[76] Davidonis G., Hinojosa O. (1994). Influence of seed location on cotton fiber development in planta and in vitro. Plant Sci..

[77] Popat R., Banakara K. (2020). Doebioresearch: R Package for Design of Experiments and Biological Research. https://cran.r-project.org/web/packages/doebioresearch/doebioresearch.pdf.

