# Supplementary material for: Carbon Dioxide Enrichment Partially Alleviates the Impact of Drought Stress on Cotton Growth and Yield
Source: Plants (Basel). 2026 Jul 17;15(14):2189. doi: 10.3390/plants15142189 (PMC13415392; doi:10.3390/plants15142189)
Supplement: Supplementary file 1 [file plants-15-02189-s001.zip › Figure S1.pdf]

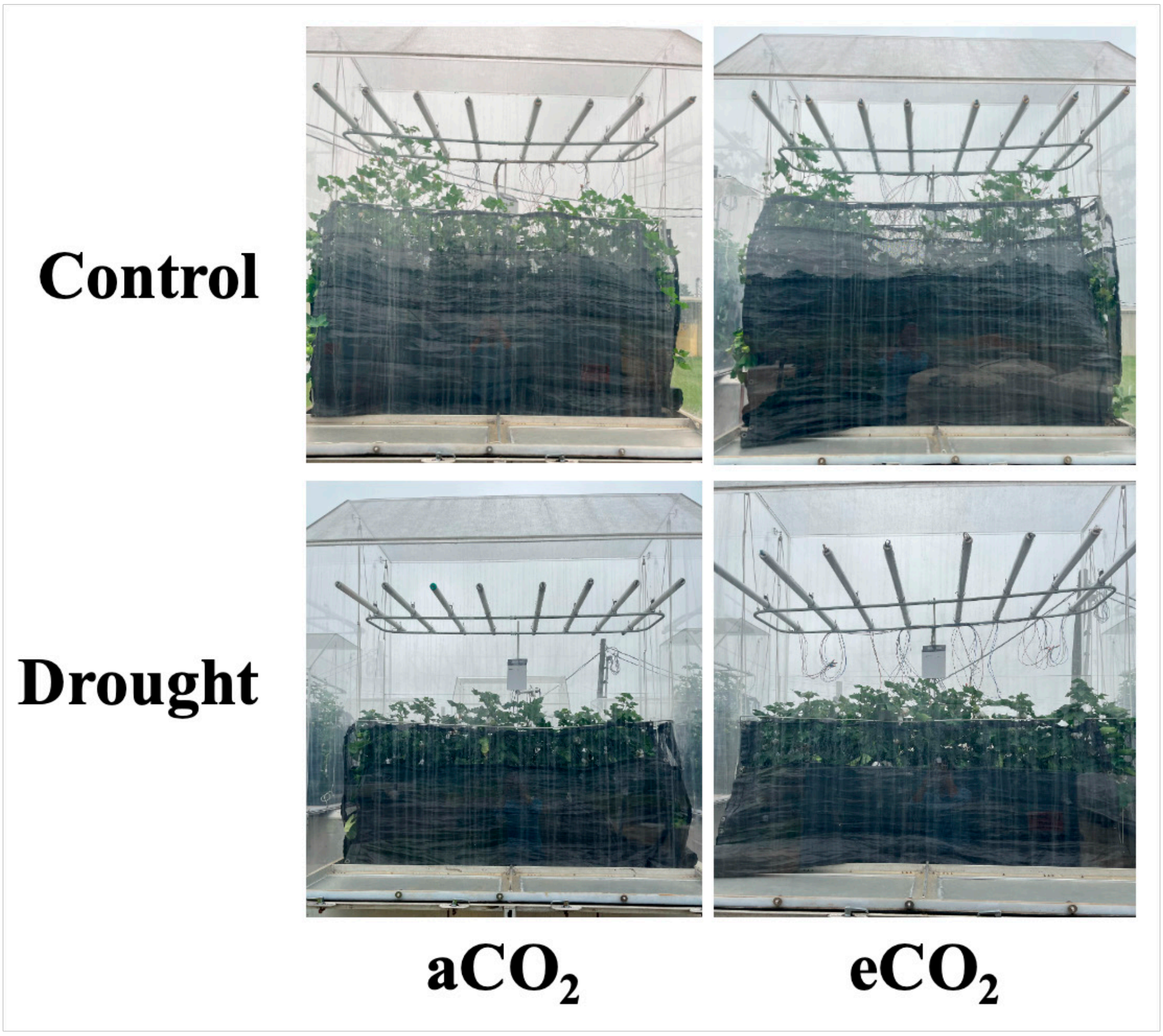

Figure S1. Cotton plants under control (well-watered, 0.12 m^3 H_2O m^{-3} volumetric soil moisture content) and drought (0.09 m^3 H_2O m^{-3} volumetric soil moisture content) conditions under two CO_2 concentrations: 425 ppm (ambient, aCO_2) and 725 ppm (enriched, eCO_2) in the Soil-Plant-Atmospheric-Research facility.
