# Supplementary material for: Carbon Dioxide Enrichment Partially Alleviates the Impact of Drought Stress on Cotton Growth and Yield
Source: Plants (Basel). 2026 Jul 17;15(14):2189. doi: 10.3390/plants15142189 (PMC13415392; doi:10.3390/plants15142189)
Supplement: Supplementary file 1 [file plants-15-02189-s001.zip › Figure S2.pdf]

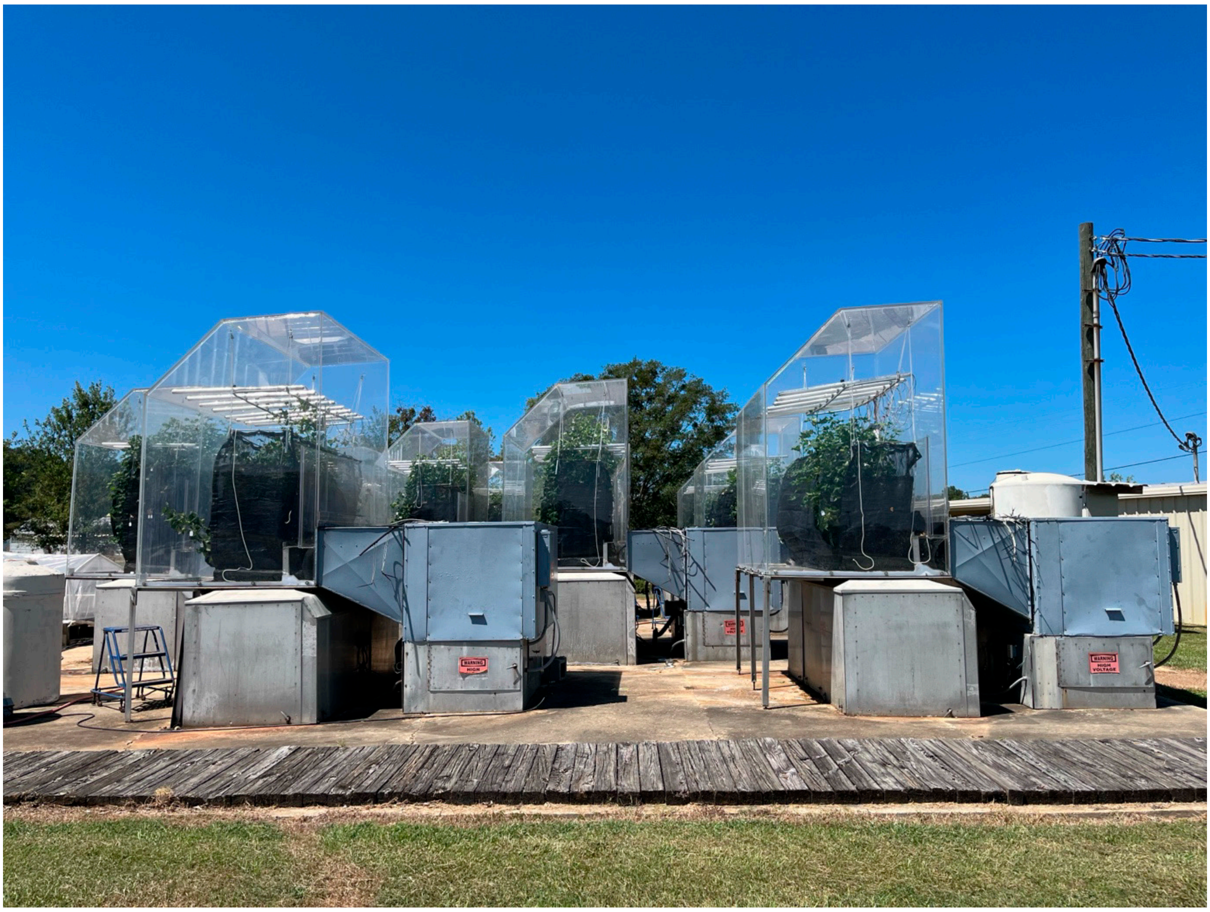

Figure S2. The Soil-Plant-Atmosphere-Research facility (SPAR) used in the study located at Environmental Plant Physiology Laboratory, Mississippi State University, MS, USA.

Each SPAR facility consists of a plexiglass chamber to hold the plant canopy and metal bin to accommodate the root system. A heating and cooling system is present at the rear side of the facility which controls the temperature inside the chamber. The CO_2 levels are monitored by a CO_2 analyzer and adjusted through the control system.
